# Everybody's Page

**Published:** 1890-06-28

**Authors:** 


### Everybody's Page.

Hippocrates said that to teach a man from his youth to preserve his health, strength, and vigour unimpaired is the grand secret worth acquiring.

What are temperance people to do when they are beset by difficulties of this kind? We noticed on a public-house the other day a board hung out with this notice: "One mile to the next public-house." Is not that a beguiling advertisement?

We have been hearing a good deal about Heligoland this week. One thing we hear is that there are 2,000 inhabitants on the island, and eight cows! From July to October 12,000 to 13,000 people visit the island. We suppose they bring their tinned milk with them.

It will be interesting to see whether the return of prosperity, which seems likely to be an accomplished fact in New Zealand, will have an appreciable effect in the consumption of spirits there. During the past ten years the decline in their consumption has been very marked; cynical people will put this down to bad times; perhaps they will be right.

It is odd to see how fashion is often retrograde. We have arrived at very perfection in the manufacture of clear plate-glass; so perfect is it that every ray of sunshine can find its way into our rooms. We are always saying how essential sunshine is to health, but Fashion just now thinks that leaded casements and tiny panes of glass and stained windows of dull green or red are old world and picturesque. Sunshine and health therefore are secondary considerations.

The Finnish Government hope by means of the steamer "Murtaja," which has been completed within the last few weeks, to keep open during the winter season the navigation between Stockholm and Hango. She does not cut through, but rides over the ice, crushing it by the sheer force of her weight, and can thus make a path through ice 25 inches thick. We shall no doubt soon hear of the Russian Government following their example, and so opening the Neva several weeks earlier than has hitherto been the case.

The Bill for the maintenance of destitute parents which Mr. Gainsford Bruce proposes is to avoid a poor old decrepit person from becoming chargeable to the parish when he has a child perfectly well able to support him. No honest person would let their father or mother go to the workhouse if able to keep them out; unfortunately, however, everybody is not honest, and often quite indifferent as to what becomes of an aged parent. On the other hand, the magistrates will have to look out for those parents who have a dislike to work, and will do their best to be "maintained" by assuming decrepitude when they are really far from it.

Somebody has written to *The Times* suggesting that the introduction of "ticket system and waiting offices" would be a great benefit to the public and prevent the fighting "like wild beasts" which is necessary to obtain a seat on an omnibus in any crowded part. The omnibuses are crowded no doubt, and the women having discovered that the outside of an omnibus is pleasant, the men do not get seats so easily nowas formerly. But we do not think the writer to *The Times* can often travel about half past five by the Underground, for he would see that the ticket system there does not enable one altogether to pick and choose one's seat in the train. No, the real grievance against the omnibus is the racing between rival companies, which is bound to end in some dreadful catastrophe ere long.

Dr. Ephraim Cutter recommends that those who suffer from insomnia should give up coffee and tea for a time, and try hot water or milk instead.

There is nothing pleasanter to use as a cure for rough or sun-burnt skin than slices of raw cucumber. It is a much cheaper remedy than any of the preparations in bottles, and the best way is to cut off a slice and rub the juice well into the skin and then dry with a towel.

Owing to the agricultural depression in Spain emigration from that country is now going on on a large scale. The Argentine Republic and Chili seem to be the spots most favoured as the new land of adoption by the emigrants, many of whom are said to show clear signs of distress.

This is the age of scientific discovery, and Dr. Malin-Conico, of Naples, is not going to be left behind others in enriching it. He has discovered the "microbe of old age," and he says it is transmitted by heredity. Somehow, after we have heard of elixirs of life, etc., Dr. Malin-Conico does not sound altogether original in this idea.

Old beliefs and customs are not so very extinct in England after all. In some parts of Worcestershire it is firmly believed that a loaf of bread baked on Good Friday, hung up in the kitchen, is a cure for diarrhœa, and that rain-drops caught and bottled on Ascension Day will cure sore eyes!

The *New York Medical Record* thinks that the lesson to be learnt from Succi's fast is that people eat too much and (in America at least) drink too little. We think that a considerable reduction in both eating *and* drinking would be the lesson a good many people in this country might profitably learn from it.

Many of us know what an uncomfortable sensation buzzing or humming in the ear is, but we hear of the case of a man who suffered from cracking noises in his ear every five or six seconds loud enough to be heard by a person standing near him. Examination of the ear proved a split in the drum to exist, and every time the man swallowed, air was forced through the split.

In spite of all that has been said about cheapness of food, vegetables are a necessary article of diet that we Londoners find are very dear and not always very fresh. Oh! you country people with your beautiful gardens, if sometimes instead of throwing your surplus cabbages and lettuces to the pigs you would send a carriage free hamper of them to one of the London hospitals, you would be sure your gift would be appreciated. The Children's Hospital at Great Ormond Street, for example, says that there is no greater boon than a present of "country vegetables."

Among the numerous works carried out by the Sisters of the Church at Kilburn is the restaurant at the docks. It supplies cheap, good meals, and acts as a counter attraction to the gin-palaces and other degrading refreshment bars. Trucks laden with good hot food are sent out from the Mission House in Dock Street, and these wait at the dock gates and supply the men as they pass by. We commend the sisters heartily for this, in that they do good and yet do not pauperise; and we think that they must often unknowingly do something for temperance, for will not the comfort of a large, really good bowl of soup for a halfpenny leave a far more lasting impression than two-pennyworth of bad gin?

